# Tumor circulome in the liquid biopsies for cancer diagnosis and prognosis

**DOI:** 10.7150/thno.40532

**Published:** 2020-03-15

**Authors:** Jicheng Wu, Shen Hu, Lihong Zhang, Jinxia Xin, Chongran Sun, Liquan Wang, Kefeng Ding, Ben Wang

**Affiliations:** 1 Cancer Institute (Key Laboratory of Cancer Prevention and Intervention, China National Ministry of Education), The Second Affiliated Hospital, Zhejiang University School of Medicine, Hangzhou 310009, China; 2 Institute of Translational Medicine, Zhejiang University, Hangzhou 310029, China; 3 Department of Obstetrics, The Second Affiliated Hospital, Zhejiang University School of Medicine, Hangzhou 310009, China; 4 Department of Biochemistry, College of Biomedical Sciences, Zhejiang University School of Medicine, Hangzhou 310009, China; 5 Department of Neurosurgery, The Second Affiliated Hospital, Zhejiang University School of Medicine, Hangzhou 310009, China

**Keywords:** liquid biopsy, tumor circulome, tumor screening

## Abstract

Liquid biopsy is a convenient, fast, non-invasive and reproducible sampling method that can dynamically reflect the changes in tumor gene expression profile, and provide a robust basis for individualized therapy and early diagnosis of cancer. Circulating tumor DNA (ctDNA) and circulating tumor cells (CTCs) are the currently approved diagnostic biomarkers for screening cancer patients. In addition, tumor-derived extracellular vesicles (tdEVs), circulating tumor-derived proteins, circulating tumor RNA (ctRNA) and tumor-bearing platelets (TEPs) are other components of liquid biopsies with diagnostic potential. In this review, we have discussed the clinical applications of these biomarkers, and the factors that limit their implementation in routine clinical practice. In addition, the most recent developments in the isolation and analysis of circulating tumor biomarkers have been summarized, and the potential of non-blood liquid biopsies in tumor diagnostics has also been discussed.

## Introduction

**Figure 1:**
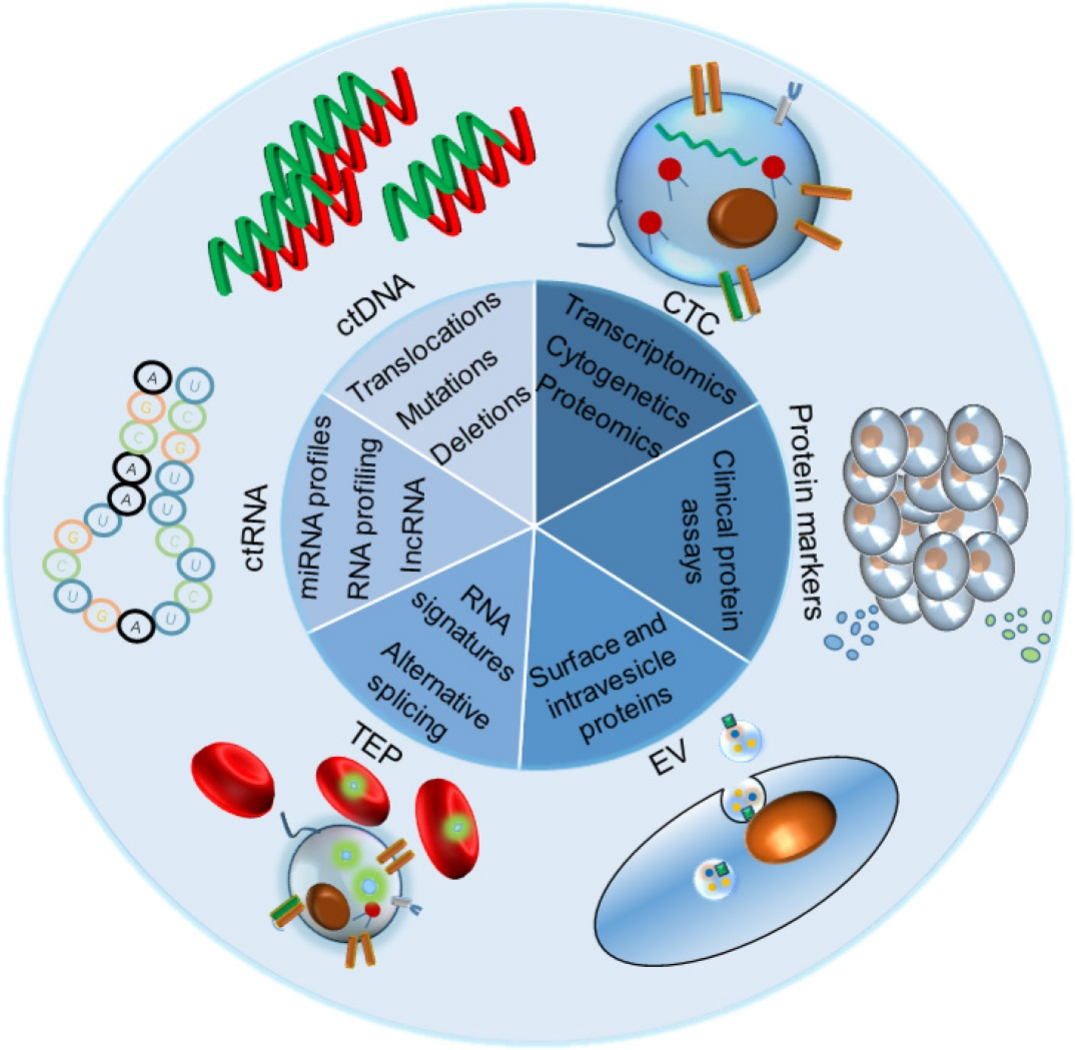
The liquid biopsy markers (LBMs) commonly used in clinical or laboratory screening include circulating tumor proteins, circulating tumor ctDNA, circulating tumor cells (CTCs), tumor-derived extracellular vesicles (EV) and their components, circulating tumor ctRNA and tumor-cultured platelets (TEPs). All of them can be used directly or indirectly for cancer screening and diagnosis.

Early detection of cancer can significantly improve the therapeutic outcomes and patient prognosis. However, large-scale screening for early stage tumors is still not possible due to lack of suitable techniques. Although tissue biopsy is the gold standard of tumor detection and diagnosis, it is limited by the difficulties in obtaining tissue samples, poor sensitivity and accuracy, high procedural costs, inability to distinguish between heterogeneous tumors and invasiveness 1, and is therefore incompatible with longitudinal clinical monitoring. Liquid biopsies on the other hand can be collected in a non-invasive manner, and have gained considerable attention in recent years for early cancer screening, tumor progression monitoring, assessing therapeutic response and clinical prognosis, and detecting recurrent and refractory tumors 2, 3. Common liquid biopsy markers (LBMs), including circulating tumor nucleic acids (ctDNA and ctRNA), circulating tumor cells (CTCs), tumor-derived extracellular vesicles (tdEVs) and tumor-educated platelets (TEPs), can be used as cancer biomarkers either directly or indirectly and have been summarized in Figure 1 4.

**Figure 2:**
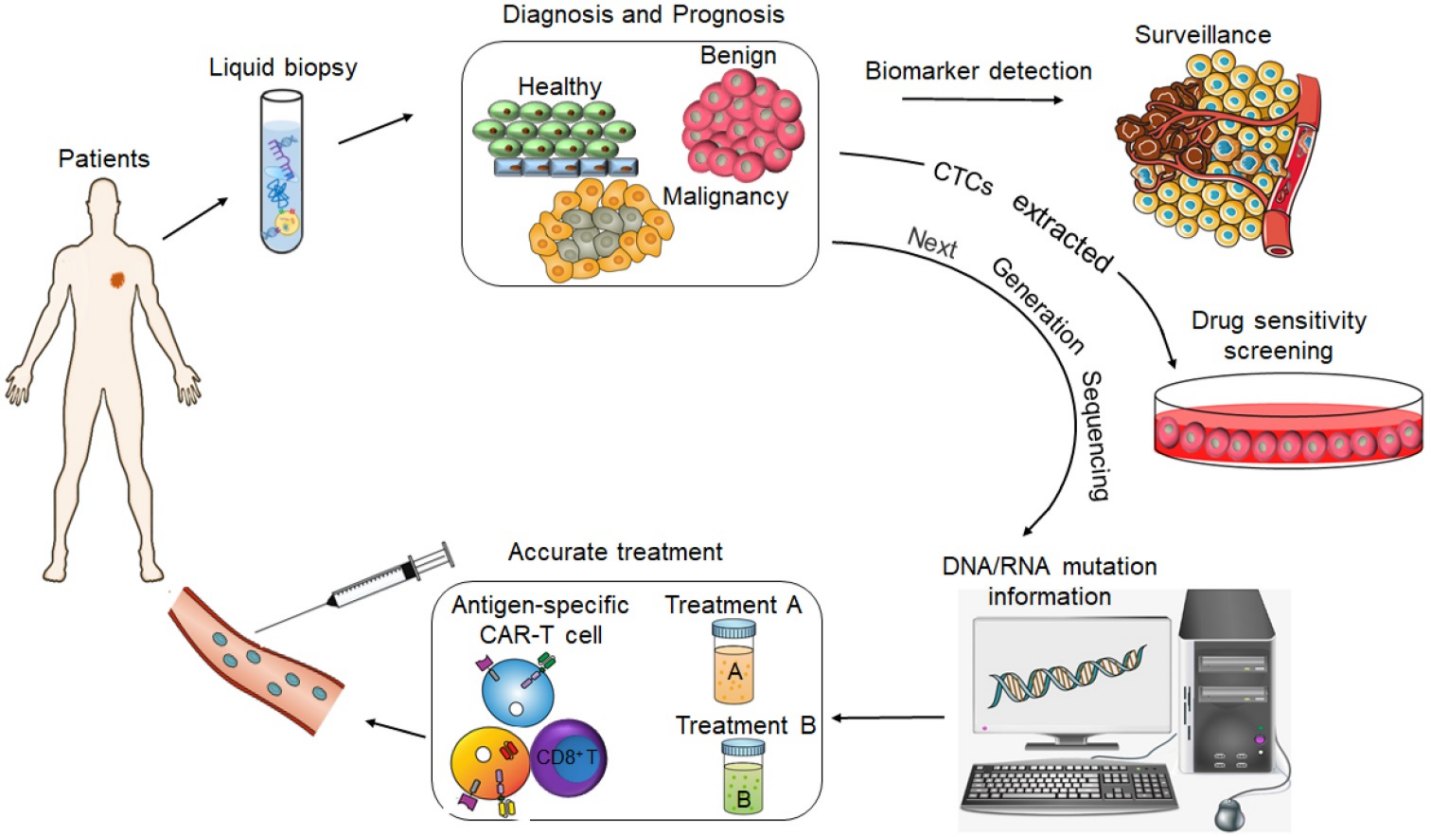
The value of liquid biopsy in tumor diagnosis and precision medicine.

CtDNA was approved by FDA as a diagnostic marker for lung cancer and for screening colorectal cancer (CRC) in 2016 5, and the CellSearch^®^ CTCs capture system has also been approved by the FDA for diagnosing metastatic prostate, breast and colon cancers 6. Although these are important milestones in the field of liquid biopsy and molecular diagnosis, such molecular-based tools have limited application in routine clinical practice since they are not approved worldwide and are not part of standard cancer diagnostics. The current focus is on developing more sensitive and high throughput early cancer diagnosis techniques, such as the mRNA Sentinel Principle Technology**^i^**, new generation sequencing technology (NGS) 7, single-cell protein analysis and sequencing 8, high-resolution flow cytometers 9 and PCR- based assays 10, in order to expand the clinical applications of liquid biopsies. In fact, the identification of LBMs in liquid biopsy specimens has emerged as a potential means to improve prognostic prediction, guide risk-adaptive or precision therapy, and detect patients at the highest risk for disease relapse (Figure 2).

In this review, we have summarized the diagnostic and prognostic value of liquid biopsies in various cancers, the recent developments in the isolation of cancer biomarkers, potential clinical applications, and the limitations. In addition, the possibility of using body fluids other than blood, such as urine, salivary, cerebrospinal fluid (CSF), stool, sputum and lacuna liquid such as pleural effusion, as tumor-derived biomarkers has also been discussed.

## Circulating protein markers

Circulating protein markers are the most established diagnostic tools for cancer, and include prostate specific antigen (PSA) for screening prostate cancer 11, cancer antigen (CA) 15-3 for postoperative follow-up of breast cancer, carbohydrate antigen 19-9 (CA 19-9), carcinoembryonic antigen (CEA) for intrahepatic cholangiocarcinoma (ICC), alpha-fetoprotein (AFP), and carbohydrate antigens for screening multiple malignancies 13, 14. However, individual protein markers are significantly limited in terms of sensitivity and specificity, and associated with high false-positive rates. A panel of multiple protein biomarkers can improve the diagnostic and prognostic accuracy by reducing the number of both false positives and false negatives 15, 16. In addition, diagnostic platforms using multiple circulating protein markers have been established in recent years for cancer screening. Matrix-assisted laser desorption /ionization time-of-flight mass spectrometry (MALDI-TOF MS) is a high-throughput, highly sensitive proteomics platform that can not only identify novel tumor-specific markers, but also enable early screening and diagnosis of tumors. Long et al. recently developed a nanoplatform for diagnosing multiple myeloma via detection of the urine Bence-Jones protein; macroporous ordered silica foams (MOSF) were firstly used to enrich the proteins and the resulting nanoparticle-protein composites were analyzed by MALDI-TOF MS. This panel diagnosed multiple myeloma with high sensitivity (95.24%, 20/21) and specificity (100%, 27/27), and is therefore a highly promising tool for the clinical diagnosis of Bence-Jones protein-related diseases 17. Park et al. also developed a MALDI-TOF MS-based total serum protein fingerprinting tool for liver cancer diagnosis and confirmed its high sensitivity and specificity 18. Taken together, diagnostic platforms based on circulating markers can be used for effective, high-throughput screening for various cancers and other diseases. These novel platforms have to be validated by testing on more clinical samples, and their performance can be further improved by extensive and reliable database matching, as well as intelligent and rapid information reporting systems.

The extracellular matrix (ECM) molecules, such as matrix metalloproteinases (MMPs), MMP- degraded collagens, collagen oligomeric matrix protein (COMP) and fibronectin, are released into the bloodstream from solid tumors, and have therefore emerged as promising surrogate markers of tumor development or clearance 19, 20. In addition, these circulating ECM proteins reflect the tumor- microenvironment crosstalk, and can provide novel insights into tumor initiation and progression 21. Each step in tumorigenesis is characterized by a distinct tumor microenvironment (TME) or tumor- associated inflammatory signature, which can affect tumor progression either favorably or adversely. Therefore, markers associated with tumor-associated inflammation or the TME can potentially increase the chances of monitoring the onset of tumor niches, metastatic growth, patient prognosis and anti-cancer drug efficacy. Lin et al. recently found that increased levels of CXCL8 and colony stimulating factor 2 (CSF-2), which facilitates macrophage-driven CXCL8 secretion, indicated poor clinical outcome and tumor progression in gastric cancer patients. Mechanistically, CXCL8 inhibits CD8^+^ T cells function by inducing the immunosuppresive PD-L1 on macrophages 22. Lundgren et al. determined that high density of CD1a^+^ dendritic cells (DCs) was an independent prognostic factor for reduced OS in pancreatobiliary cancer, and increased prevalence of CD68^+^ and CD163^+^ tumor- associated macrophages (TAMs) were significantly associated with poor OS in periampullary adenocarcinoma patients 23. The results indicate that markers associated with tumor inflammation and the microenvironment can be used for noninvasive diagnosis, screening, and postoperative follow-up of cancer.

## Circulating tumor nucleic acids

Circulating or extracellular tumor nucleic acids are secreted from primary or metastatic cancer cells following apoptosis or necrosis 24, and differ significantly from the ctDNAs/RNAs in the sera of healthy individuals. Interestingly, the ctDNA isolated from the blood of patients with pancreatic cancer harbors the mutated KRAS gene 25, and N-ras gene mutations have been detected in the plasma of myelodysplastic syndrome and acute myelogenous leukemia (AML) patients 26, which further underscores the utility of ctDNA as a cancer biomarker. Several studies have subsequently reported the presence of extracellular-DNA (exDNA) in sera/plasma from patients with different cancers 27-30. Similarly, the presence of exRNA, including messenger RNAs (mRNAs), microRNAs (miRNAs) and long non-coding RNAs (lncRNAs), have also gained attention in recent years as potential non-invasive cancer biomarkers 31. The altered ctRNA levels in cancer patients often return to normal following surgery, and therefore are suitable indicators of therapeutic response 32, 33. As a means of liquid biopsy, cfDNA/ctDNA technology can be further divided into two approaches: detection of gene mutations and methylation. The COBAS^®^ EGFR Mutation Test v2 (Roche Diagnostics), the first ctDNA-based diagnostic test used in clinics, was approved by FDA in 2017 for monitoring the response to epidermal growth factor receptor (EGFR)-tyrosine kinase inhibitors (TKIs) in non-small cell lung cancer patients harboring EGFR-TKI sensitizing mutations 34. In addition, EpiproColon^®^, a FDA-approved screening test for CRC, is based on the methylation pattern of the SEPT9 gene promoter 35. However, Getz et al. recently suggested that detecting gene mutations in liquid biopsies may have limited use for early screening of cancer, since normal tissues also harbor mutations and somatic variations in multiple genes. They found that 95% of individuals have somatic mutations in at least one tissue, while 33% carry cancer-related mutations. Therefore, the methylation status of cfDNA/ctDNA is a better marker for early cancer screening, with greater sensitivity and specificity 36. Besides, when using genetic mutations or molecular markers for tumor screening and diagnosis, genetic mutations or molecular markers expression in some special diseases, such as clonal erythropoiesis, need to be considered independently, as these diseases have mutations at specific genetic loci, or express a special molecular marker which may mislead the results when screening for molecular cancer-specific data onto liquid biopsy 37.

MicroRNAs are a class of small non-coding RNAs that regulate gene expression and are critical in several biological and pathological processes, including cancer initiation and progression. Several preclinical studies have reported circulating miRNA as tissue-specific cancer biomarkers that can not only monitor a prognosis but also determine the origin of tumor metastases 38, 39. In addition, serum microRNA levels can also reflect other physiological conditions such as pregnancy, and can even be used to determine the pregnancy stage 40.

Currently, ctDNA analyses are based on PCR or next-generation sequencing (NGS). Allele-specific PCR was the earliest approach used in ctDNA detection 41, and a modified version is the technical basis of the COBAS^®^ EGFR test 34. More sensitive PCR technologies, such as digital PCR (dPCR), droplet digital PCR (ddPCR), and beads, emulsion, amplification and magnetics (BEAMing) have been developed in recent years and increase the accuracy of ctDNA detection 42. Despite the high sensitivity, rapidity and relative low costs, these PCR techniques are limited by low multiplexing capacity since they can only analyze a restricted number of loci simultaneously rather than entire gene sequences. Compared to PCR-based technologies, NGS has lower sensitivity due to even fewer number of loci that it can analyze. Furthermore, since the mutant allele fraction (MAF) in a given locus is greater than 5%, whole- exome sequencing (WES) has the lowest sensitivity in analyzing ctDNA sequences compared to the other techniques 43. The sensitivity of NGS can be increased by including patient or cancer-specific gene panels, e.g. the cancer personalized profiling deep sequencing (CAPP-Seq) technology 44. In addition, the background signals can be minimized by tagging each template sequence with unique molecular identifiers (UMIs), and using selective nucleases on the non-mutated DNA 45. In addition, artificial intelligence based on whole-genome sequencing (WGS) is a recent breakthrough in ctDNA analysis through liquid biopsies. In a more recent study, Velculescu et al. developed the artificial intelligence platform DELFI (DNA evaluation of fragments for early interception) for cancer specific screening, monitoring and diagnosis. They used known tumor-specific mutations to label tumor-derived ctDNA, compared them with the homologous healthy free DNA and determined the fragment length distribution, followed by introducing all information into the database. After establishing this intelligent platform, other variables such as GC abundance, position of chromosome arms, mutant alleles etc. were also introduced and each was assigned a specific score to improve detection. The information collated in the intelligent platform and the database can then be used to quickly and sensitively distinguish healthy patients from cancer patients on the basis of their ctDNA profile. This platform was used to test the plasma samples of 236 cancer patients and 245 healthy individuals, and showed respective accuracy rates of 91% and 98%. Thus, artificial intelligence platforms highlight the clinical importance of cell-free DNA and provide a proof-of-principle approach for the screening, early detection and monitoring of human cancer 46.

However, ctDNA detection has several limitations, such as low sensitivity at the early stages of cancer. The incipient tumors shed very low levels of ctDNA, which decrease the MAF and may escape detection 44. To eliminate individual differences due to hereditary predispositions and improve the detection efficiency, the liquid biopsies should be collected as close to the tumor as possible, which unfortunately is technically challenging 47. In addition, healthy individuals should also be screened for cancer-associated mutations to determine whether a particular mutation has a predictive value in early detection 42. CancerSEEK, a recently developed high throughput screening test for liquid biopsies, can detect ctDNA in the blood of cancer patients with high sensitivity 48.

## Circulating tumor cells

During malignant progression, the primary tumor masses shed a significant number of cells that invade adjacent tissues, and migrate to distant sites to establish metastatic clones and also regenerate blood vessel walls to support neo-angiogenesis 49. These circulating tumor cells (CTCs) are therefore a reliable biomarker of cancer metastasis. A number of clinical trials, which mostly using the Cellsearch platform established prognostic and predictive value in patients, have confirmed that changes in CTC count between baseline and the second anti-cancer treatment coursed in advanced breast cancer 50 had been associated with an adverse prognostic and predictive value on the patient's outcome, with a reported good “negative predictive value” of CTCs. This finding has been reiterated in other metastatic cancer types, such as colorectal 51, prostate 52 and ovarian 53 cancers. Monitoring CTCs can not only improve the chances of early cancer detection and identify novel drug targets, but also predict patient prognoses and therapeutic responses. In addition, the CTC load can stratify patients into different risk groups for (neo) adjuvant therapies 54, 55. CTCs are extremely rare in the peripheral blood of cancer patients, usually one per million blood cells. Therefore, it is technically challenging to detect them with high sensitivity and specificity. In addition, CTC isolation currently depends on the surface expression of epithelial markers; for instance, the CellSearch^®^ system captures epithelial cells from the blood using EpCAM-coated magnetic beads, which are then identified using fluorescently-labeled antibodies against cytokeratins (CK 8, CK 18, CK 19). There are several hitherto non-approved systems as well that detect stem-like, mesenchymal-like and hybrid CTC subpopulations, which are clinically significant since CTCs show partial or complete epithelial- mesenchymal transition (EMT) and some even acquire stem cell-like characteristics 56, 57. Therefore, the conventional epithelial marker-dependent CTC detection can neither distinguish between these subpopulations, nor can it identify the origin of micrometastatic and metastatic CTCs. Therefore, further research on these CTC subpopulations can provide new insights for anti- cancer therapeutics 58. *Ex vivo* expansion of CTCs from individual patients can enable personalized drug screening, and assist in making more effective treatment decisions based on the unique tumor mutation profiles and drug sensitivity patterns 59. Certainly, although *in vitro* culture of CTCs has important guiding value for clinical tumor treatment, this approach is fraught with several limitations. For instance, pre-sample processing, enrichment and sorting, and improper culture conditions can cause irreversible damage to the CTCs. Furthermore, long- term *in vitro* culture and multiple passages may alter the CTCs genetically and epigenetically, such that they no longer represent the phenotype of the original tumor. Therefore, it is essential to develop a more effective CTC sorting and enrichment system, as well as improve the *in vitro* culture conditions. In addition, establishing co- cultures of CTCs with immune cells and other blood cells can improve our understanding of the survival mechanism of CTCs in the peripheral blood. This in turn can help develop new intervention strategies and further promote the clinical usage of CTCs as an important tool for liquid biopsy. The isolated CTCs can be genetically analyzed using qPCR, dPCR-based mutational spectroscopy technology, target NGS and genome-wide sequencing technologies 60. In addition, cytogenetic techniques such as fluorescence in situ hybridization (FISH) can be used to identify tumor-specific chromosomal rearrangements in the CTCs 61. Since CTCs are involved in tumor progression and initiate metastases, mutiomics analyses at the single-cell level can help dissect the complex relationships between the tumor subpopulations and the surrounding normal tissue. Thus, single CTC genomics and transcriptomics are invaluable to the study of tumor heterogeneity and for comparative analysis with tissue biopsies 62-64.

CTC enrichment and detection methods are broadly classified as biological, physico-chemical and functional based on the specific CTC properties that are utilized. The biological methods of capturing CTCs depend on the expression of surface biomarkers; for example, CellSearch^®^ is based on enriching the EpCAM^+^ cells 50 and can be combined with other cancer biomarkers or CD45^+^ depletion 65. In addition, surface immuno-phenotyping can be synergized with microfluidics to further enhance CTC yields, such as the CTC-Chip 66 and NanoVelcro 67 platforms. CTCs can also be separated from normal blood cells based on their size, density, and dielectric properties through filtration 68, microfluidics 69, differential centrifugation 70, densitometry (MagDense) 71 and di-electrophoresis (DEPArray: a semiautomated system that allows the isolation of rare cells) 72. Functional CTC captures assays include Vita-Assay^TM^ 73, EPISPOT^®^ assay 74 and TelomeScan^®^ 75 that respectively exploit CAM digestion, protein release during culture and telomerase expression. All these methods have their own advantages but due to the rarity, vulnerability and heterogeneity of CTCs, any one method cannot capture sufficient number of CTCs for clinical and other applications. Therefore, a combination of two or more methods may improve CTC enrichment for liquid biopsies.

## Tumor-derived extracellular vesicles (tdEVs)

Chargaff and West discovered back in 1946 that removal of the pelleted plasma fraction after high- speed centrifugation inhibited plasma clotting 76. Years later, Peter Wolf determined that small (20-50 nm) lipid-bilayer-enclosed structures or vesicles that extruded from the platelets were the anti-coagulation factors 77. Subsequent studies reported that the transferrin receptors on reticulocytes interacted with vesicles secreted from these cells 78-80. Extracellular vesicles (EVs) are membranous granules released from all types of cells under physiological and pathological conditions, as well as in response to proteases, inflammatory cytokines, growth factors, biomechanical shear, stress-inducing factors and apoptotic signals. Based on their biogenesis, content and secretory pathways, EVs can be divided into exosomes and microvesicles 81. Exosomes are exfoliated vesicles with ecto-enzyme activity and were first described by Trams et al. 82. They originate during endocytic internalization from the inter 9nal budding of the plasma membrane. The early endosomes fuse with the Golgi complex to form late endosomes that then give rise to intraluminal vesicles contained within multi vesicular bodies (MVBs). The latter either fuse with the plasma membrane to release exosomes through exocytosis or are degraded upon fusing with the lysosomes 83.

EVs, especially the exosomes, mediate cell-cell communication by transporting cargo like proteins, DNA, mRNAs and miRNAs from the donor to target cells 84, 85. For example, exosome-mediated glia-neuron communication maintains neuronal integrity 86. Exosomes had increasingly gained attention as messengers of cancer cells wherein they can reprogram the transcriptome of target cells by transporting regulatory RNAs. For example, breast cancer cell-derived exosomes harbor components of the RISC (RNA-induced silencing complex)-loading complex (RLC) including pre-miRNAs, Dicer, Argonaute2 and TRBP (Trans-activation of transponder RNA binding proteins), and have a cell-independent capacity to process precursor miRNAs (pre-miRNAs) into mature miRNAs. The RISC-loaded exosomes are known to trigger the malignant transformation of the adjacent normal breast epithelial cells and drive tumor progression 87. Studies show that cancer cells release significantly higher amounts of EVs compared to non-malignant cells, and are therefore suitable diagnostic markers as well as anti-cancer therapeutic targets 88-90. Tumor-derived extracellular vesicles (tdEVs) not only influence the growth, progression and drug resistance of the parent tumors by altering the immediate microenvironment, but also create favorable conditions in distant organs to allow growth of disseminated tumor cells, a process known as pre-metastatic niche (PMN) formation 91. For instance, metastatic melanomas release EVs that carry programmed death-ligand 1 (PD-L1) on their surface, which suppresses the function of CD8 T cells and facilitates tumor growth 92. Furthermore, the distinct integrin expression patterns on tdEVs determine their adhesion to specific cell types and ECM proteins in specific organs 93.

The proteins and RNAs present in the lumen and membrane of EVs are potential cancer biomarkers that can allow early tumor diagnosis. Typical exosome- enriched proteins include members of the tetraspanin family (CD9, CD63 and CD81), members of the endosomal sorting complexes required for transport (TSG101 and Alix), and heat-shock proteins (Hsp60, Hsp70 and Hsp90) 94. In addition, tumor-associated proteins such as CEA, EGFR VIII, HER2 and MelanA, along with a range of DNA, mRNAs and miRNAs have also been detected in exosomes. The exosomes harboring cancer-specific proteins like CD63, CD9 and CD81, and microRNA signatures have been isolated from the blood of breast cancer 95, ovarian cancer 96 and glioblastoma patients 97, and from the urine of prostate cancer patients 98, 99. Exosomal miRNAs are protected from degradation of RNAse unlike the naked circulating miRNAs, and are therefore more reliable diagnostic biomarkers of tumor type and stage 100. One study showed that compared to healthy controls, lung adenocarcinoma patients harbored twelve unique exosomal miRNAs (miR-17-3p, miR-21, miR-106a, miR-146, miR-155, miR-191, miR-192, miR-203, miR-205, miR-210, miR-212, and miR-214). In addition, four exosomal miRNAs (miR-378a, miR-379, miR-139-5p, and miR-200b-5p) were able to distinguish patients with lung carcinoma from healthy former smokers with 97.5% sensitivity and 72% specificity, and six miRNAs (miR-151a-5p, miR-30a-3p, miR-200b-5p, miR-629, miR-100 and miR-154-3p) could differentially diagnose between lung adenocarcinoma and granuloma 101. Another study reported 80.65% and 83.33% sensitivity, and 91.67% and 90.32% specificity of exosomal miRNAs for diagnosing lung adenocarcinoma and squamous cell carcinoma (SCC) respectively. Taken together, these circulating miRNAs are potentially sensitive, non-invasive biomarkers for early NSCLC diagnosis 102. Therefore, exosome-based assays have gained considerable attention for cancer diagnosis. For instance, peripheral blood-derived exosome screening is far less invasive compared to colonoscopy, and more specific compared to carcinoembryonic antigen (CEA) or carbohydrate antigenic determinant (CA 19-9) for diagnosing colon cancer.

EVs are released by several cell types, including B and T lymphocytes, dendritic cells, mast cells, intestinal epithelial cells, neurons, tumor cells and MSCs 103-108, and are present in physiological fluids such as urine, plasma, cerebrospinal fluid, milk and various exudates 98, 109-114. However, although tumor-derived EVs (tdEVs) are promising blood biomarkers for cancer disease management, blood is a highly complex fluid that contains multiple particles of the same size range as tdEVs, which obscures their unimpeded analysis. Therefore, a highly sensitive and specific approach is required to capture and detect tdEVs. The isolation strategies of EVs are primarily based on their physical and biological properties. Almost all EVs, especially exosomes, can be extracted from body fluids by normal density-gradient centrifugation, ultracentrifugation, and the more advanced EV array and immuno-bead precipitation 90, 115. Exosomes typically had diameters ranging from 30 to 100 nm and density 1.13-1.19 g/ml, and are usually saucer-shaped or hemispherical with a concave side 116, 117. Although high exosome yield and purity can be achieved with filtration, the high pressure generated during the process can damage their structural integrity 118. Therefore, immuno-affinity captured with antibodies targeting the surface proteins of EVs such as the tetraspanins are preferred for naive and intact EVs. This approach also allows high purity isolation of distinct immuno-phenotypic EV subsets. Beekman *et al*. 119 had recently developed a multi-modal analysis platform for the specific capture of tdEVs on antibody-functionalized stainless-steel substrates, followed by their analysis using SEM, Raman spectroscopy and AFM at the single EV level in terms of size and size distribution, and chemical fingerprint. A single surface marker however can miss significant subpopulations, which may be obviated by targeting multiple markers via microchip-based *in situ* immunoassay 120. Microfluidics have emerged as a promising new method for rapidly capturing EVs based on their physical or biochemical features 121, and can boost affordable EV-based medical diagnostics. Size-exclusion chromatography (SEC), polymer precipitation, magnetic nanopore-sorting platform and alternating current electrokinetic (ACE) chip are some other techniques that have been tested for EV isolation, but are limited by low efficiency and purity. As already mentioned, the EVs can be profiled for somatic mutations, splice variants, gene fusions and aberrant gene or protein expression by RT-PCR, NGS, Western blotting, ELISA etc. However, these methods analyze the bulk EV population and therefore disregard the inherent heterogeneity. The micro-flow cytometry platform however can detect single circulating exosomes using beads 122, and nanoparticle tracking analysis (NTA) enables high resolution protein analysis of these individual EVs 123. Several approaches have been examined to analyze the heterogeneity (DNA, RNA or proteins) of different exosomes and clarify their tissue of origin. We developed a robust microchip-based method for the selective and quantitative analysis of exosomes using digital detection integrated with nucleic acid amplification 124. Kamali-Moghaddam et al. 125 employed a proximity-dependent barcoding assay to simultaneously profile multiple surface proteins on individual exosomes. This detection platform combines surface immuno-phenotyping with digital detection or NGS technology, and can further enhance the precision of heterogeneity analysis, which broadens its applications in basic research as well as disease diagnosis.

Compared to ctDNA and CTCs, the clinical translation of EVs is hampered due to the technically challenging isolation methods, low analytical sensitivity and poor stability. On the other hand, the amount of circulating EVs is significantly higher compared to that of CTCs 126, and can be indicative of the existence of tumors. For instance, circulating exosome levels increase in breast and pancreatic cancer patients 127, and the number of circulating microparticles (MPs) is significantly higher in multiple myeloma (MM) patients compared to healthy individuals. Moreover, the circulating MP level is also a potential marker for the diagnosis and prognosis of advanced NSCLC 128. Due to the lack of unified standards for sample processing as well as EV separation and analysis, the clinical applications of EVs as a method of liquid biopsy are limited 129. Another factor limiting EV-based diagnostics is the lack of standardized guidelines for defining EV characteristics, as well as the lack of appropriate controls for validation. The International Extracellular Vesicle Association has recently upgraded a comprehensive series of guidelines and recommendations to redefine EVs nomenclature 130, which will subsequently facilitate EV research and redefine the gold standard for EV extraction and analysis, and therefore expedite their clinical translation.

In addition to proteins, DNA, glycans, lipids and metabolites are also present on the surface of EVs, and are potential biomarkers for EV enrichment 131. A database of EV-associated RNAs or proteins can greatly increase the chances of identifying novel circulating biomarkers specific for different cancer types. The exact role of EVs in various pathophysiological conditions also needs to be established in order to develop a standardized EV diagnostic system.

## Tumor-educated platelets

Calverley et al. were the first to observe significant differences in the platelet genomic profiles of cancer patients and healthy individuals. They found that 197 platelet-associated genes were down- regulated in patients with metastatic lung cancer, and multiple genes were also spliced differentially between the control and patient groups 132. Subsequently, Nilsson et al. reported that platelets from cancer patients can actively absorb tumor-derived EVs and take up RNA from tumor cells 133. Therefore, these tumor-educated platelets (TEPs) are also potential biomarkers for cancer diagnosis and screening, and have been the focus of recent studies 134. In 2015, Best et al. sequenced the transcriptomes of platelets derived from cancer patients in order to determine the diagnostic potential of TEPs by mRNA sequencing 135, and identified patients with six types of localized or metastatic tumors and healthy individuals with 96% accuracy, and the primary tumor location with 71% accuracy. The same group applied particle-swarm optimization (PSO)-enhanced algorithm to select possible biomarker panels from platelet RNA-sequencing libraries, and achieved accurate TEP-based detection of early- and late-stage NSCLC 136. This algorithm may also enable optimization of diagnostics using other liquid biopsies. However, although many detection platforms for TEPs have been developed, patient and control samples ought to be collected under the same conditions to minimize any deviations. This is especially significant for platelet-related tests since they can be easily activated and alter their transcriptome profiles. In addition, exosome and cfDNA profiling can also be greatly affected by differences in sample collection and handling. Taken together, TEPs are potential biomarkers for the early diagnosis, screening and therapeutic monitoring of cancers, and their clinical translation would depend on developing appropriate isolation techniques.

## Other biological specimens as liquid biopsies

**Figure 3:**
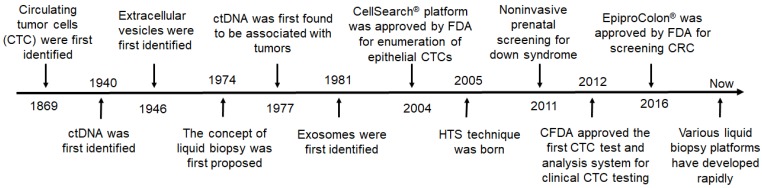
The milestones in the development of liquid biopsies. HTS: high throughput sequencing; CRC: colorectal cancer; FDA: Food and Drug Administration; CFDA: China Food and Drug Administration.

It has been 150 years since Ashworth first discovered CTCs, and now various liquid biopsy platforms and techniques have been developed for screening, detection, and diagnosis of tumors (Figure 3). During this period, various milestones of new discoveries or new technologies have greatly promoted the practical development of liquid biopsy.

**Figure 4:**
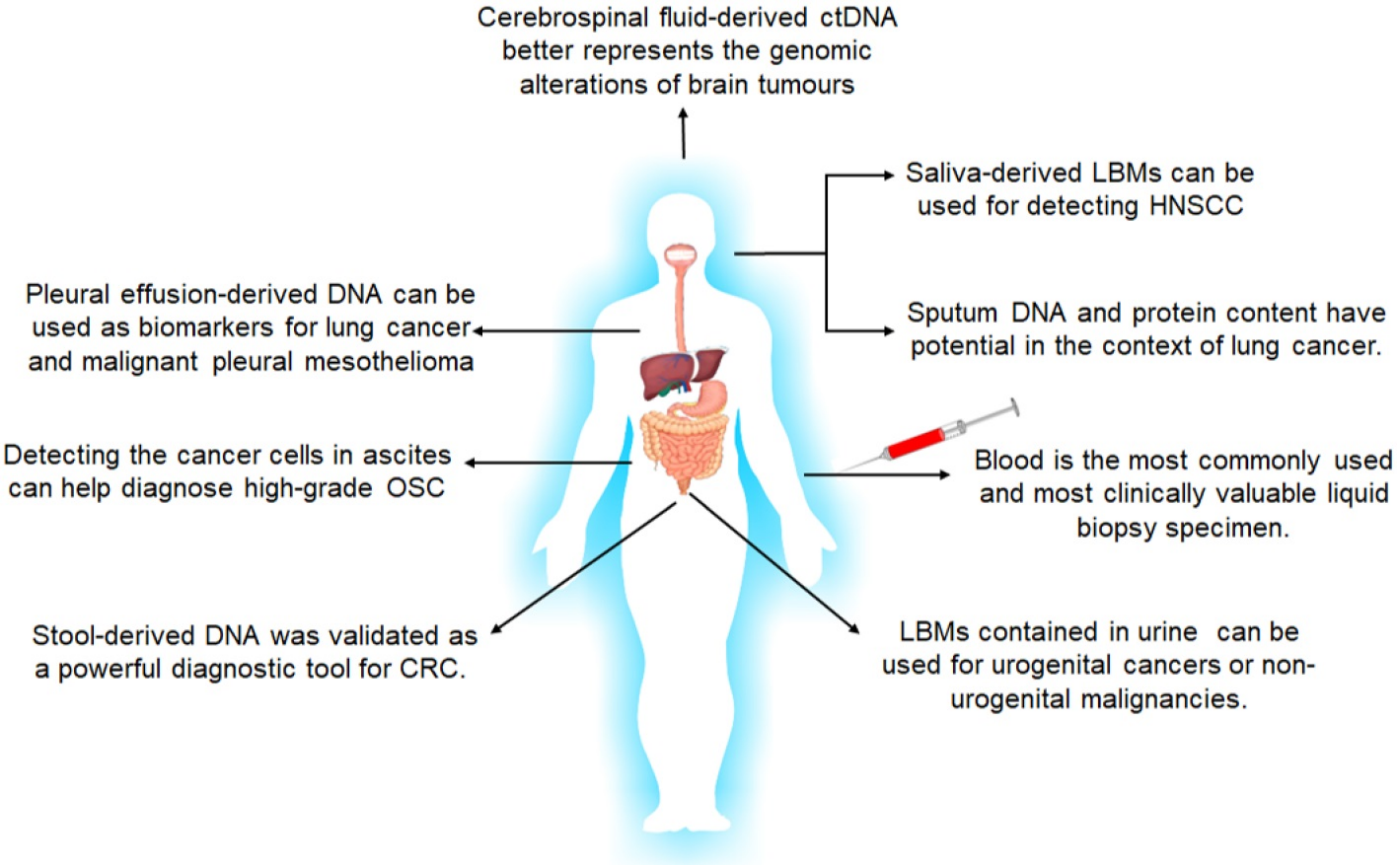
The body fluids suitable for liquid biopsies and their applications in tumor diagnosis and screening. LBMs: liquid biopsy markers; HNSCC: head and neck squamous cell carcinoma; OSC: Ovarian Serous Carcinoma; CRC: colorectal cancer.

However, although liquid biopsy typically refers to utilization of blood samples, almost all body fluids, including but not limited to urine, saliva, sputum, feces, cerebrospinal fluid and lacuna liquid such as pleural effusion, are reliable cancer biomarkers suitable for liquid biopsies 137-139 (Figure 4). Since cancer biomarkers are typically expressed in the local tumors at the early stages of cancer, they usually do not appear in circulation, which further underscores the diagnostic significance of local liquid biopsies.

For genitourinary tumors such as prostate, the bladder and cervical cancer, urine is the ideal liquid biopsy for early diagnosis and treatment, and can be easily collected and analyzed for biomarker proteins, ctDNAs or EVs. Progensa® PCA3 Assay was approved by FDA in 2012^ii^ for repetitive prostate biopsies in case of a negative first diagnosis 140. ExoDx, SelectMDx and Michigan Prostate Score (MiPS) were recently developed as non-invasive screening tools for prostate cancer diagnosis and prognosis, and primarily detect androgen-related transmembrane protease serine 2 (TMPRSS2-ERG) or second chromosome locus associated with prostate-1 (SChLAP1) in the urine 141, along with PSA levels in the blood. In addition, the ExoDx^®^ Prostate (IntelliScore)**^iii^** test can detect the expression levels of three exosome-associated RNAs in the urine with high sensitivity and specificity. Thus, urine analysis can obviate the need for invasive prostate tissue biopsies and digital rectal examination.

Salivary biomarkers include ctDNA, miRNAs and EV-associated miRNAs, and have significant clinical value in diagnosing head and neck squamous cell carcinoma and early oral cancers 142, 143. Due to the presence of the blood-brain barrier, CSF is an important source of circulating biomarkers for CNS-restricted cancers. Ding et al. 144 reported that CSF-derived ctDNA can better reflect genetic alterations in brain tumors compared to ctDNA in the bloodstream. Akers et al. 144 also found that the altered miRNA profile of CSF is predictive of gliomas. The analysis of stool-derived DNA was recently validated as a powerful diagnostic tool for CRC 146, while sputum DNA and protein content have diagnostic potential in lung cancer 147. Furthermore, presence of TP53-mutated cancer cells in ascites is indicative of high-grade serous ovarian carcinomas (HGSOCs) 148, and pleural effusion-derived DNA is a biomarker for lung cancer and malignant pleural mesothelioma 149. Therefore, local liquid biopsies have greater diagnostic value compared to peripheral blood in some cancers, and further developments in the isolation and diagnostic techniques using these fluids will greatly improve early diagnosis of tumors.

## Summary and future directions

Liquid biopsies provide a cost-effective, fast, reproducible and non-invasive source for early cancer diagnosis and prognostic monitoring. In addition, analysis of circulating tumor-derived factors or the tumor circulome in the liquid biopsies can capture the clonal heterogeneity of these tumors unlike tissue biopsies. Various liquid biopsy samples can be combined to improve the chances of cancer diagnosis, and sequential real-time biopsies will further aid in the early identification of therapy-resistant tumors. Furthermore, detection and characterization of minimal residual disease after initial therapy can also be improved by analyzing liquid biopsies. Automated chip-based devices are particularly fitting for the high throughput analysis of biomarkers from whole blood and other body fluids, and obviates time consuming and costly purification steps. However, the lack of standardized pre-analytical and analytical variables is a significant limitation in this field, and has stymied large-scale clinical applications of liquid biopsies. In conclusion, liquid biopsies can be a powerful tool for cancer diagnosis, monitoring, prognosis and individualized treatment, and can completely change the current paradigms of cancer management. However, considerable research and development are still needed to improve the isolation, enrichment and downstream analysis of circulating biomarkers.

## Abbreviations

FDA: food and drug administration
LBMs: liquid biopsy markers
CTCs: circulating tumor cells
ctDNA: circulating tumor DNA
tdEVs: tumor- derived extracellular vesicles
ctRNA: extracellular vesicles
ctRNA: circulating tumor RNA
TEPs: tumor- educated platelets
NGS: next generation sequence
PSA: prostate-specific antigen
CA: cancer antigen
CA19-9: carbohydrate antigen 19-9
CEA: carcinoembryonic antigen
MMPs: matrix metalloproteinases
COMP: collagen oligomeric matrix protein
lncRNAs: long non-coding RNAs
EGFR: epidermal growth factor receptor
dPCR: digital PCR
ddPCR: droplet digital PCR
WES: whole-exome sequence
UMIs: unique molecular identifiers
FISH: fluorescence in situ hybridization
PMN: pre-metastatic niche
PD-L1: programmed death-ligand 1
ASGPR1: asialoglycoprotein receptor-1
SEC: size-exclusion chromatography
ACE: alternating current electrokinetic
NTA: aanoparticle tracking analysis
TMPRSS2-ERG: androgen-related transmembrane protease serine 2
SChLAP1: second chromosome locus associated with prostate-1
MiPS: michigan prostate score
